# Arts on prescription for wellbeing in adults: systematic review

**DOI:** 10.3389/fpubh.2026.1833798

**Published:** 2026-06-01

**Authors:** Érica Frade Sá, Matilde Monteiro-Soares, Maria José Ribas, Annalisa Banzi, Joana Simões Henriques, Domingos Loureiro, Luís Monteiro, Sofia Baptista

**Affiliations:** 1 Faculdade de Medicina da Universidade do Porto, Porto, Portugal; 2 RISE-Health, Faculdade de Medicina da Universidade do Porto, Porto, Portugal; 3 Escola Superior de Saúde da Cruz Vermelha Portuguesa – Lisboa, Lisboa, Portugal; 4 CrossI&D – Lisbon Research Center, Lisboa, Portugal; 5 Family Health Center Garcia de Orta, Porto, Portugal; 6 CESPEB - University of Milan-Bicocca, Milan, Italy; 7 MAAT- Museum of Art, Architecture and Technology, Lisboa, Portugal; 8 Faculdade de Belas Artes da Universidade do Porto, Porto, Portugal; 9 i2ADS - Research Institute of Art, Design and Society, Porto, Portugal; 10 Department of Medical Sciences, University of Aveiro, Aveiro, Portugal; 11 Hospital CUF, Porto, Portugal

**Keywords:** anxiety, art programmes, arts on prescription, depression, mental health, social prescribing, wellbeing

## Abstract

**Introduction:**

Arts on Prescription is a social prescribing model in which health professionals refer adults to community-based artistic activities led by artists or musicians, with the goal of promoting wellbeing and mental health. This systematic review aimed to investigate whether Arts on Prescription improves wellbeing and reduces symptoms of depression and anxiety.

**Methods:**

PubMed, PsycINFO and Cochrane CENTRAL were searched in November 2024, following a previously developed search strategy. The selection of studies, quality assessment and data extraction were carried out independently by two authors. Disagreements were resolved by consensus. We included randomised control trials, quasi-experimental studies, observational studies and mixed-methods studies assessing the impact of art programmes prescribed by a health professional on adults’ wellbeing and symptoms of depression and anxiety. A narrative synthesis of the results was carried out.

**Results:**

Of the 3,561 unique citations obtained, six studies met the inclusion criteria. Analysis of the reference lists of the included studies revealed two additional pertinent studies. Five of the studies were quasi-experimental studies and three were observational studies. The sample size ranged from 12 to 1,297 participants, with an average age ranging from 43 to over 80 years old. In all studies, an improvement in wellbeing was reported following participation in Arts on Prescription programmes. Evidence regarding depression and anxiety was limited to one study, which reported statistically significant but clinically modest reductions in both outcomes.

**Discussion:**

Arts on Prescription programmes were consistently associated with improvements in wellbeing across a range of populations and settings. Preliminary evidence suggests potential benefits for depression and anxiety that warrant investigation in more rigorous study designs. Further studies are needed to overcome the limitations of the analysed studies, such as the lack of control groups, small and non-representative samples, and short follow-up periods.

**Systematic review registration:**

https://www.crd.york.ac.uk/PROSPERO/view/CRD42024572685, identifier CRD42024572685

## 1 Introduction

Interest in the arts and their effects on individuals’ health and wellbeing has been a relevant topic in the scientific community in recent years, leading to a significant increase in studies in this area since the beginning of the century. The arts are a promising tool for improving mental and physical health, with potential benefits at different stages of life (1).

Social prescribing is the process by which individuals are referred by health and social care professionals for non-clinical interventions to support their wellbeing (2). This involves referral to a link worker, whose role is to develop an intervention plan based on the preferences and values of the person and the community in which they live. The link worker will also introduce them to the intervention to be implemented (3). In this way, a link has developed between primary health care and community support, with the common goal of benefiting the individual’s health and improving the network of social relationships (4). In addition to referrals via the link worker, referrals can also be made directly by a general practitioner (GP) or by the individuals themselves (3). Social prescribing provides a non-medical referral option that can be used to complement existing treatments (5).

Arts on Prescription (AoP) was established in the mid-1990s as one of the earliest models of social prescribing (6). AoP Stockport began in 1994 as the first programme aimed at improving the wellbeing of patients with mild to moderate depression (2). AoP programmes are led by artists or musicians and involve people from the community (2). Examples of these activities include painting, sculpting, and visiting museums. Patients are referred to participate in artistic activities, usually once or twice weekly, lasting around 2 h, for 6 to 12 weeks (7).

Simon Opher evaluated the cost–benefit impact of Artlift, a programme funded by the Gloucestershire National Health Service (NHS). The evaluation found that participation in arts interventions led to a reduction in GP visits and in associated NHS healthcare costs (8). This type of intervention could have benefits not only for the individuals referred, but also for the economy and healthcare spending. It is worth noting that art therapy and AoP are different. Art therapy aims to support psychotherapy or emotional expression, whereas AoP emphasises creative exploration and process rather than skill acquisition (7).

Central to the World Health Organization’s definition of health, wellbeing can be defined as a dynamic state in which individuals are able to realise their potential, work productively and creatively, form positive relationships, and contribute to their community. Wellbeing is strengthened when people are able to achieve their personal and social goals while feeling that they have a purpose in society (9). Additionally, in 2019, around 970 million people worldwide were living with a mental disorder, including 301 million with anxiety disorders and 280 million with depressive disorders. Given their high prevalence and the consequences these diseases can have on individuals’ lives, including their effects on interpersonal relationships, work capacity, quality of life, and economic costs (10, 11), there is a need to look for new strategies to provide better support to patients (11).

We therefore aimed to systematically review and synthesize the existing evidence on the efficacy of AoP (the intervention) in reducing levels of depression and anxiety and improving wellbeing (the outcome) in adults (the population) compared with any other strategy.

## 2 Methods

We conducted this systematic review in accordance with the Preferred Reporting Items for Systematic Reviews and Meta-Analyses (PRISMA) guidelines (12). Our protocol was registered in the International Prospective Register of Systematic Reviews (PROSPERO; CRD42024572685).

### 2.1 Data sources and searches

A search strategy was developed to enable a systematic, efficient, and sensitive search for articles (Supplementary file 1). We used free text and database-specific keywords for the terms “Arts on Prescription,” “depression,” “anxiety,” and “wellbeing,” and adapted them to the different databases. Articles identified in the reference lists of the articles included in this review were analysed manually by two authors to find additional articles in accordance with the inclusion and exclusion criteria.

PubMed, PsycINFO, and Cochrane CENTRAL (Cochrane Central Register of Controlled Trials) were searched in November 2024 using the specified search strategy.

### 2.2 Study selection

All citations were imported into Rayyan (13). After importing the citations into the platform, the Rayyan software was used to identify duplicates and to support the independent screening process by the authors.

Articles were analysed and selected independently by two authors according to previously defined inclusion and exclusion criteria, based on the title and abstract and subsequently on the full text. Disagreements were resolved by consensus.

We included articles that met the following criteria: (i) randomised controlled trials (RCTs), quasi-experimental studies, observational studies and mixed-methods studies; (ii) an adult population (≥18 years); (iii) intervention involving arts programmes prescribed by any health professional; (iv) outcomes including changes in patients’ levels of depression, anxiety, and wellbeing measured by validated scales or questionnaires. We excluded articles that were: (i) published in 1990 or earlier; (ii) studies in which the intervention consisted of the prescription of art as an adjunct to other therapies. No language restrictions were applied. We accepted the standard of care or any other intervention as a comparator strategy. Only studies published from 1990 were included, since AoP programmes became established in the mid-1990s. Previous studies were excluded because it is thought that they may not adequately reflect the properly structured intervention that is intended to be studied.

Mixed-methods studies were eligible for inclusion according to the same criteria. For the analyses, we focused exclusively on the quantitative component of such studies, corresponding to the study designs defined in the inclusion criteria (RCTs, quasi-experimental studies, and observational studies). This approach is consistent with established practice in systematic reviews that include mixed-methods studies (14, 15).

Articles in which the intervention was art therapy—a type of psychotherapy that involves an art therapist working with individuals or small groups, using visual and tactile media as a way to express themselves (2, 16)—were excluded.

We contacted the authors of articles for which full texts were unavailable, or in which only a protocol was identified, to determine whether the respective study had been completed and published.

### 2.3 Data extraction, quality assessment, and data synthesis

Data extraction was performed independently by three authors, with two authors assigned to each article. The data to be extracted were pre-defined and included study author, publication year, study design, country (and city), total sample size, sample size by group, loss to follow-up, mean age and standard deviation (SD), gender (number and percentage of females), referral process, intervention type, intervention duration, control group or comparator, outcome measurement methods (including reporting format), follow-up time, changes in patients’ levels of depression, anxiety, and wellbeing, other outcomes, missing outcome data, and any other relevant data. Any disagreements arising during this process were resolved by consensus.

Two authors analysed the articles and determined the study design according to their characteristics, reaching consensus. We used the Joanna Briggs Institute (JBI) critical appraisal tools to evaluate the quality of the studies according to their design: RCTs (17), quasi-experimental (18), or observational (19) studies, as appropriate. These JBI checklists are specific to the different study designs and comprise multiple assessment parameters recorded as “yes,” “no,” “unclear,” or “not applicable,” with space for comments. This assessment was performed independently by three authors, with two authors assigned to each article. Discrepancies were resolved by consensus.

A narrative synthesis was then performed. Data were presented in a table organised according to the different topics extracted. The results were also presented in different sections according to the effect of AoP: wellbeing, anxiety, depression, and other relevant effects.

## 3 Results

### 3.1 Study selection

**Figure 1:**
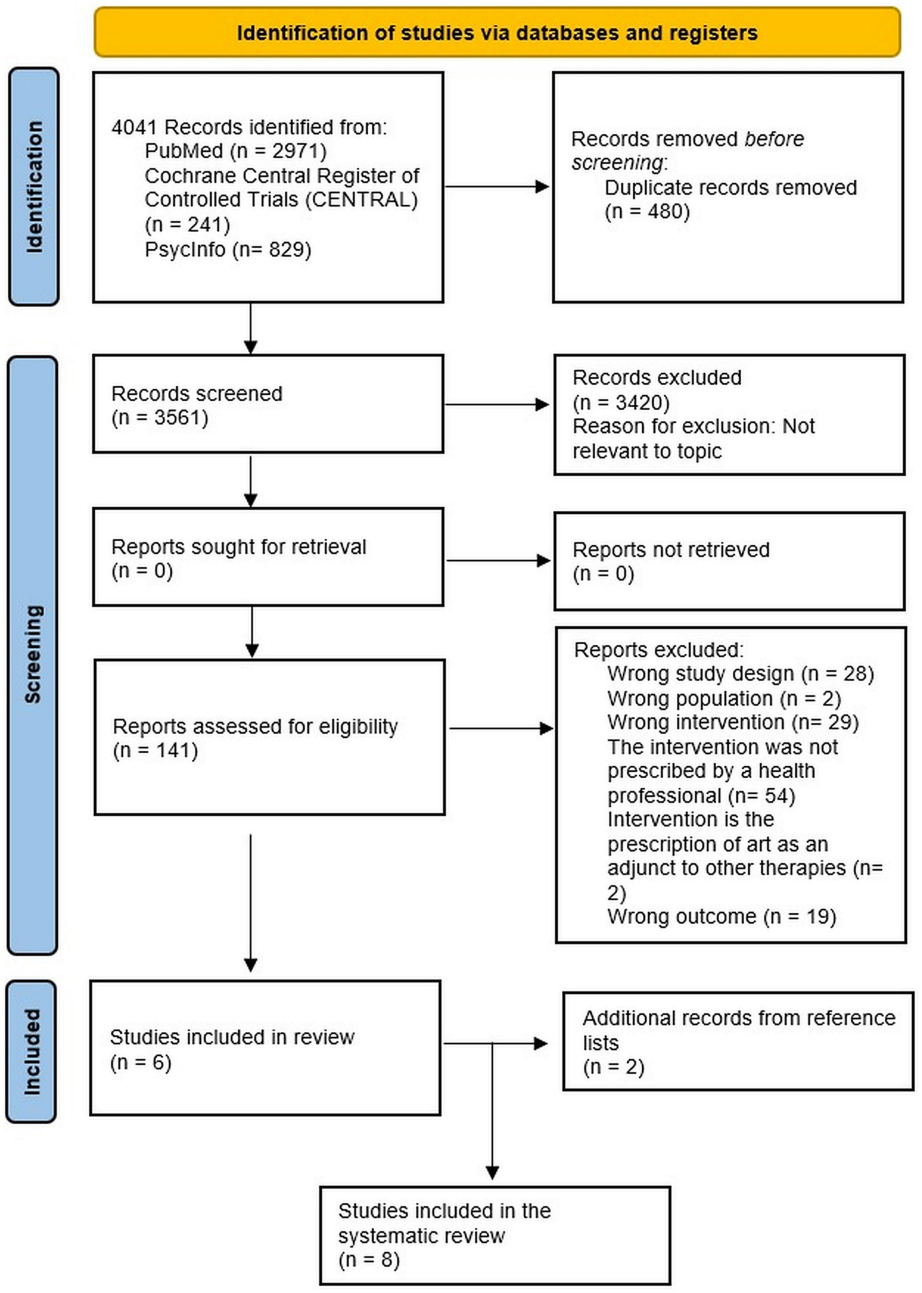
PRISMA flow diagram.

The PRISMA flow diagram (Figure 1) illustrates the search process. Following the search of the databases, 4,041 articles were identified, of which 480 were duplicates and were deleted. Thus, 3,561 articles were analysed. Of these, 3,402 were excluded based on the title and abstract, according to the inclusion and exclusion criteria. In addition, 7 protocols that corresponded to previously selected studies, 2 protocols from unpublished studies, and 9 protocols/studies for which the author did not respond were excluded. Thus, 141 articles were analysed in the second phase, based on the full text. Of these, 134 were excluded, and 7 were included.

Upon analysing the reference lists of the included articles, we identified two additional articles that met the inclusion and exclusion criteria. Three of the articles included are part of the same programme (20–22), and one of them (20) reports preliminary results from the cohort analysed in another study (21), which followed the cohort over a longer period. Therefore, both articles (20, 21) were analysed together. In total, nine articles, corresponding to eight studies, were included in our systematic review.

### 3.2 Assessment of risk of bias

The quality of the articles included in this review was assessed using the JBI critical appraisal tools. The results are presented in Table 1.

**Table 1:** Quality assessment.

Author(s) (reference)	JBI critical appraisal checklist for cohort studies											
	Question 1	Question 2	Question 3	Question 4	Question 5	Question 6	Question 7	Question 8	Question 9	Question 10	Question 11	Total
Crone et al. (20, 21)	No	Yes	Yes	Yes	No	Not applicable	Yes	Yes	No	No	Yes	6/11
Sumner et al. (22)	Not applicable	Not applicable	Yes	Yes	Yes	Not applicable	Yes	Yes	Yes	No	Yes	7/11
Holt et al. (27)	Not applicable	Not applicable	Yes	No	No	Not applicable	Yes	Yes	No	No	Yes	4/11

	JBI critical appraisal checklist for quasi-experimental studies									
	Question 1	Question 2	Question 3	Question 4	Question 5	Question 6	Question 7	Question 8	Question 9	Total
Thomson et al. (23)	Yes	No	Yes	Not applicable	Wellbeing - No	Wellbeing - Yes	Wellbeing - Yes	Wellbeing - Yes	Wellbeing - Yes	6/9
Poulos et al. (28)	Yes	No	Yes	Not applicable	Wellbeing - No; Levels and frequency of creativity - No; Frailty - Yes	Wellbeing-Yes; Levels and frequency of creativity - Yes; Frailty - Yes	Wellbeing - Yes; Levels and frequency of creativity - Yes; Frailty - Yes	Wellbeing - No; Levels and frequency of creativity - Yes; Frailty - Yes	Wellbeing - Yes; Levels and frequency of creativity - Yes; Frailty - Yes	5/9
Van de Venter et al. (24)	Yes	No	Yes	Not applicable	Wellbeing - No	Wellbeing - Yes	Wellbeing - Yes	Wellbeing - Yes	Wellbeing - Yes	6/9
Thomson et al. (25)	Yes	No	Yes	Not applicable	Wellbeing - Yes	Wellbeing - Yes	Wellbeing - Yes	Wellbeing - Yes	Wellbeing - Yes	7/9
Vogelpoel et al. (26)	Yes	No	Yes	Not applicable	Wellbeing - No	Wellbeing - Yes	Wellbeing - Yes	Wellbeing - No	Wellbeing - Yes	5/9

The mean quality score for the three cohort studies was 5.7 out of 11 (JBI cohort tool), with the main sources of bias being: incomplete follow-up (JBI item 9), absence of strategies to address missing data (item 10), and limited identification and control of confounding factors (items 4–5).

The mean quality score for the five quasi-experimental studies was 5.8 out of 9 (JBI quasi-experimental tool), and the main sources of bias were: the absence of a control group (item 2), lack of multiple outcome measurements (item 5), and absence of strategies to address missing data (item 8).

The mean quality score for the study analysing anxiety and depression was 7 out of 11 (JBI Cohort tool), with the main source of bias was the absence of strategies to address incomplete follow-up (item 10).

### 3.3 Study characteristics

The characteristics of the studies are presented in Table 2 and Supplementary file 2. The studies were published between 2014 and 2021. Seven of the studies (88%) were conducted in the United Kingdom (20–27) [four of these were specifically conducted in England (20–22, 25, 27)]. One of the studies (13%) was conducted in Australia (28).

**Table 2:** Study characteristics.

Author(s) (reference)	Study year	Study design	Country (city)	Sample size (total and by group) (*N*)	Follow-up losses (*N*)	Age (years), mean (SD)	Gender (female), *N* (%)
Crone et al. (20, 21)	2018 (DC 2009–2016)	Observational	England (Southwest)	1,297. Attenders (referred and attended): 818 (completed: 651). Non-attenders (referred but did not attend): 440.	39	51.1 (15.87)	980 (77.0)
Thomson et al. (23)	2020 (DC February–April 2018)	Quasi-experimental, sequential mixed methods	United Kingdom	20. NA	0	53 (44–70)	N*
Poulos et al. (28)	2019	Quasi-experimental, based on mixed methods	Australia (Sydney)	127. NA	20	78.1 (7.99)	94 (74.0)
Van de Venter et al. (24)	2014	Quasi-experimental, based on mixed methods	United Kingdom	44. NA	0	43 (27–73)	36 (82)
Sumner et al. (22)	2021 (DC 2017–2019)	Observational	England (Southwest, Gloucestershire)	245. NA	0	50.5 (15.71)	196 (80)
Thomson et al. (25)	2018 (DC 2015–2017)	Quasi-experimental, based on mixed methods	England (Central London and Kent)	115. NA	0	65–94	72 (63)
Vogelpoel et al. (26)	2014	Quasi-experimental, based on mixed methods	United Kingdom	12. NA	4	Mean age of over 80 (61–95)	9 (75)
Holt NJ (27)	2020 (DC September 2017–July 2019)	Observational	England (Bristol)	66. NA	0	47 (25–75)	58 (88)

Author(s) (reference)	Intervention type	Intervention duration/follow-up time (in weeks)	Control group or comparator	Changes in patients’ levels of depression
Crone et al. (20, 21)	Led artist, activities: poetry, ceramics, drawing, mosaic and painting; Groups 3–10.	8 or 10	B/A	NA
Thomson et al. (23)	Led by a horticultural specialist, an art tutor and a museum volunteer. Activities: Combination of outdoor horticultural activities and indoor nature-based creative activities. Weekly.	10	B/A	NA
Poulos et al. (28)	Led by professional artists with the support of a community care worker or volunteer. Courses: visual arts, photography, dance and movement, drama, singing and music. Groups: 6–8.	8–10	B/A	NA
Van de Venter et al. (24)	Led artist. Activities: painting, textiles, music, photography and film.	20	B/A	NA
Sumner et al. (22)	Activities: visual arts (painting, ceramics, mosaics, photography) or performing arts (playwrighting, creative writing, singing). Weekly.	8	B/A (both initial referral and re-referral)	Method: Self-reported; Instrument: PHQ-8; Results: ↓ (initial referral: 13.4 ± 6.46 vs. 11.5 ± 6.45, *p* < 0. 001; re-referral: 13.2 ± 6.23 vs. 10.7 ± 6.21, *p* < 0. 001; across both cycles: 14.0 ± 6.37 vs. 11.28 ± 6.07, *p* < 0.001). In the multimorbid subgroup, depression also ↓ (initial referral: 14.3 ± 6.00 vs. 11.7 ± 6.10, *p* < 0.001; re-referral: 13.9 ± 6.12 vs. 10.8 ± 5.57, *p* = 0.002; across both cycles: 14.5 ± 6.29 vs. 11.2 ± 5.36, *p* = 0.001).
Thomson et al. (25)	Led by museum staff and volunteers. Activities: curator talks, behind-the scenes tours, object handling and discussion, and art activities inspired by the exhibits.	10	B/A	NA
Vogelpoel et al. (26)	Led by visual and tactile arts facilitators, supported by sense support staff and communicator guides.	12	B/A	NA
Holt NJ (27)	Led by skilled arts and health practitioners. Activities: art workshops. Weekly.	Thirty participants attended a 12-week programme, and thirty-six completed two programmes.	B/A	NA

Author(s) (reference)	Changes in patients’ levels of anxiety	Changes in patients’ levels of well-being	Outcome missing data, *N* (%)
Crone et al. (20, 21)	NA	Method: Self-reported; Instrument: WEMWBS; Results: ↑ (who attended: 38.1 ± 9.59 vs. 44.6 ± 9.84, *p* < 0.001; who were engaged: 38.0 ± 9.61 vs. 44.6 ± 9.79, *p* < 0.001; multimorbidity subsample: 36.7 ± 9.94 vs. 42.8 ± 9.32, *p* < 0.001; all categories of participation: 37.8 ± 9.63 vs. 44.4 ± 9.98, *p* < 0.001).	0 (0).
Thomson et al. (23)	NA	Method: Self-reported; Instrument: UCL Museum Wellbeing Measure, specifically the positive generic wellbeing measure; Results: ↑ (16.70 vs. 25.30, *p* < 0.001). Each of the individual mood items also ↑.	0 (0).
Poulos et al. (28)	NA	Method: Self-reported; Instrument: WEMWBS; Results: ↑ (mean increase: 6.86, 95% CI: 5.33–8.38, *p* < 0.001).	17 in wellbeing data (percentage not available).
Van de Venter et al. (24)	NA	Method: Self-reported; Instrument: WEMWBS; Results: ↑ (B/A: 38.2 vs. 46.2; after a mean of 14 sessions: mean increase 8.0, 95%CI: 4.8–11.2, *p* < 0.0001).	0 (0).
Sumner et al. (22)	Method: Self-reported; Instrument: GAD-7; Results: ↓ (initial referral: 11.9 ± 6.00 vs. 9.6 ± 5.80, *p* < 0.001; re-referral: 11.7 ± 5.87 vs. 9.5 ± 6.02, *p* < 0.001; across both cycles: 11.8 ± 6.05 vs. 9.9 ± 6.02, *p* = 0.008). In the multimorbid subgroup, anxiety also ↓ (initial referral: 12.0 ± 5.99 vs. 9.8 ± 5.33, *p* = 0.001; re-referral: 11.7 ± 5.77 vs. 9.5 ± 5.81, *p* = 0.010; across both cycles: 12.1 ± 6.44 vs. 9.8 ± 5.74, *p* = 0.036).	Method: Self-reported; Instrument: WEMWBS; Results: ↑ (initial referral: 37.1 ± 9.71 vs. 41.9 ± 10.40, *p* < 0.001; re-referral: 38.6 ± 9.92 vs. 42.4 ± 10.16, *p* < 0.001; across both cycles: 37.4 ± 9.77 vs. 42.3 ± 10.17, *p* < 0.001). In the multimorbid subgroup, wellbeing also ↑ (initial referral: 37.3 ± 8.84 vs. 43.0 ± 8.89, *p* < 0.001; re-referral: 38.4 ± 9.94 vs. 42.6 ± 10.12, *p* = 0.001; across both cycles: 36.6 ± 8.6 vs. 42.4 ± 10.11, *p* < 0.001).	0 (0).
Thomson et al. (25)	NA	Method: Self-reported; Instrument: MwM-OA; Results: All emotions ↑ from pre- to post-session (start-programme: *F*(1,88) = 72.228, *p* < 0.001, partial eta squared = 0.451; mid-programme: *F*(1,83) = 67.651, *p* < 0.001, partial eta squared = 0.449; end-programme: *F*(1,76) = 54.689, *p* < 0.001, partial eta squared = 0.418)	0 (0).
Vogelpoel et al. (26)	NA	Method: Self-reported; Instrument: WEMWBS; Results: mean score ↑ (from 41 to 47; positive gains were made in 11 of the 14 areas).	0 (0).
Holt NJ (27)	NA	Method: Self-reported; Instrument: WEMWBS; Results: ↑ (first programme: mean of 5.01 units; second programme: mean of 4.73)	Not specified.

B/A: before versus after design. DC: data collection. GAD-7: generalised anxiety disorder scale. MwM-OA: museum wellbeing measure for older adults. NA: not applicable. PHQ-8: patient health questionnaire eight-item version. ↑: increase. ↓: decrease. * similar. In mixed-method studies, we focused our analysis on the quantitative phase. Referral process for all studies: Referral person.

Although our analysis focused on the quantitative phase of the studies, only three of them employed exclusively quantitative methods (20–22, 27) and were observational studies. Five studies adopted a mixed-methods approach (23–26, 28), and their quantitative components were designed as single-arm quasi-experimental studies. In all studies, the sample underwent AoP and the outcomes were compared before and after the intervention.

The sample size ranged from 12 to 1,297 participants. The mean age of participants ranged from 43 to over 80 years old. Seven studies had a predominance of female participants (20–22, 24–28), while one study (23) had a similar number of participants of each sex. In three studies (20, 21, 26, 28), loss to follow-up was reported (between four and 39 participants).

In all studies, patients were referred by a healthcare professional who considered that the patients could benefit from the intervention and had the necessary conditions to participate. The interventions included a range of activities such as poetry, ceramics, drawing, mosaics, painting, textiles, photography, film, dance, movement, drama, singing, music, playwriting, and creative writing. Two of the studies involved a museum-based intervention (23, 25), one of which combined outdoor horticultural activities with indoor, nature-based creative activities (23).

Programmes were led by artists (20, 21, 24, 28), horticultural specialists, art tutors, and museum volunteers (23), museum staff and volunteers (25), visual and tactile arts facilitators (26), and skilled arts and health practitioners (27). One study (22) did not provide information about who led the intervention. Only two studies (20, 21, 28) provided information on the number of participants that could participate in each programme [3–10 participants (20, 21) and 6–8 participants (28)].

The intervention duration and follow-up times varied across studies: 8 weeks (22), 10 weeks (23, 25), 8 or 10 weeks (20, 21, 28), 20 weeks (24), and 12 weeks (26, 27). One of these studies allowed participants to take part in two programmes (27).

Wellbeing was measured in all studies. Six studies (20–22, 24, 26–28) used the Warwick–Edinburgh Mental Wellbeing Scale (WEMWBS) (29, 30), one study (23) used the UCL Museum Wellbeing Measure, specifically the Positive Generic Wellbeing Measure (31, 32), and one study (25) used the Museum Wellbeing Measure for Older Adults (MwM-OA) (31, 32). One study (22) measured anxiety symptoms using the Generalised Anxiety Disorder Scale (GAD-7) (33). One study (22) measured depression symptoms using the eight-item Patient Health Questionnaire (PHQ-8) (34).

Levels and frequency of creativity, and measures of frailty criteria, were also assessed in one study (28). Individual mood was self-reported using the Short Mood Scale (SMS) (35, 36) in one study (27). All of the studies measured outcomes before and after the intervention, and only one reported missing data (28). Two studies (21, 22) analysed these measures in a multimorbid subgroup.

### 3.4 The effect of arts on prescription on wellbeing

Wellbeing increased in all studies (20–28), as reported in the “Changes in patients’ levels of wellbeing” section of Table 2. The effect of AoP on wellbeing was evaluated, using one of three tools: the WEMWBS (20–22, 24, 26–28), the UCL Museum Wellbeing Measure, specifically the Positive Generic Wellbeing Measure (23), and the MwM-OA (25).

Significant increases in wellbeing were reported over time (*β* = 0.85; 95% confidence interval (CI) 0.45–1.25; *p* < 0.001), with scores improving at first programme (mean increase: 5.01), at second programme (mean increase: 4.73) and across both programmes (*p* < 0.001) (27). Significant increases was also reported in the other study (22) across both referral cycles (*p* < 0.001). However, no difference in the increase in mean WEMWBS scores was found according to the number of courses attended (28).

Two studies (21, 22) analysed a subsample of participants with multimorbidity and found a significant increase in WEMWBS scores [(21): 36.7 ± 9.94 vs. 42.8 ± 9.32, *p* < 0.001; (22) at initial referral: 37.3 ± 8.84 vs. 43.0 ± 8.89, *p* < 0.001; (22) at re-referral: 38.4 ± 9.94 vs. 42.6 ± 10.12, *p* = 0.001; (22) across both referrals: 36.6 ± 8.6 vs. 42.4 ± 10.11, *p* < 0.001], though this increase was more modest than that observed in the total sample (21).

The results obtained were influenced by the characteristics of the programme. Crone et al. (20, 21) reported that participants in the eight-week programme experienced a greater increase in WEMWBS scores (8-week programme: 37.8 ± 9.18 vs. 43.9 ± 9.65, *p* < 0.001; 10-week programme: 38.6 ± 10.19 vs. 45.7 ± 10.62, *p* < 0.001), were more likely to complete the programme, and demonstrated greater engagement than those in the 10-week programme. The impact of patients having a broader choice of intervention was also analysed. Those referred under these conditions were more engaged, however no statistically significant differences in the rate of change in wellbeing were observed (after the change: 38.3 ± 8.95 vs. 44.6 ± 9.65, *p* < 0.001; before the change: 37.5 ± 10.34 vs. 44.4 ± 10.56, *p* < 0.001).

The impact of the intervention is also influenced by individual factors. Those who completed the intervention or were classified as engaged reported higher baseline WEMWBS scores and differences between those who attended and others (by occupation, mean number of referral reasons, length of the referral course, and reason for referral) and between those who engaged and others (by occupation and mean number of referral reasons) were observed (20, 21).

The increase was slightly greater for women (women: 8.1, 95% CI 4.3–12.0; men: 5.7, 95% CI 0.4–10.8) and for Black and Minority Ethnic participants (Black and Minority Ethnic participants: 9.8, 95% CI 0.3–19.5; White British participants: 6.4, 95% CI 2.4–10.5), and slower among participants with lower baseline scores (24).

33% of the variation in WEMWBS scores could be explained by gender, ethnicity, baseline scores, and the number of sessions attended (24). In fact, the expected increase in wellbeing scores was not experienced by all individuals (with varying increases, maintenance, and decreases in WEMWBS scores over time), but the variation in slopes was not statistically significant (*β* = 0.35; 95% CI 0.05–2.28; *p* = 0.29) (27). However, a relatively low level of wellbeing was observed in one study (26), with a mean score of 47 compared to 49.9 in the general population in Scotland.

Thomson et al. (23) analysed global changes in the UCL Museum Wellbeing Measure scores and changes in each individual mood item assessed by participants using this tool. The results showed a significant increase for each mood item, with no significant differences between them. In another study (25), all emotions also increased significantly from pre- to post-session at the start, mid- and end-programme. Significant increases at items “Excited” (23), “enlightened” and “absorbed” (25) were observed. However, Vogelpoel et al. (26) observed that aspects such as “feeling optimistic,” “dealing well with problems”, and “feeling good about oneself” were assessed as slightly lower at the post-intervention evaluation than at the pre-intervention evaluation. In addition, the authors reported that the largest positive change for the group was “feeling more relaxed”, as this increased from “rarely” to “some of the time”.

In one study (25), multivariate analysis of variance (MANOVA) showed a highly significant main effect of programme, session, and emotion. A significant interaction between session and emotion was observed, but not between the programme and session, programme and emotion, or programme, session, and emotion when evaluated simultaneously. Comparing the start- and mid-programme measures, a highly significant difference between pre-session wellbeing scores (*p* < 0.002) and between post-session wellbeing scores (*p* < 0.036) was observed, but no significant differences were observed between mid- and end-programme.

### 3.5 The effect of arts on prescription on anxiety

The effect of AoP on anxiety was evaluated in one study (22). The results are presented in Table 2 (“Changes in patients’ levels of anxiety” section). The Generalised Anxiety Disorder Scale (GAD-7) was used to measure anxiety levels at the beginning and end of each referral cycle through self-reporting. These assessments were then compared between pre- and post-intervention at initial referral, re-referral, and across both referrals.

A decrease in anxiety symptoms was observed in all comparisons (at initial referral: 11.9 ± 6.00 vs. 9.6 ± 5.80, *p* < 0.001; at re-referral: 11.7 ± 5.87 vs. 9.5 ± 6.02, *p* < 0.001; across both referrals: 11.8 ± 6.05 vs. 9.9 ± 6.02, *p* = 0.008). The minimal clinically important difference (MCID) for the GAD-7 (four points) was not met, and significant differences were observed in the clinical categorisation of the scales (*p* < 0.001; scores ≥5, ≥10, and ≥15 indicating mild, moderate, and severe anxiety symptoms, respectively).

This study analysed a multimorbid subgroup and observed a decrease in anxiety symptoms across all comparisons (at initial referral: 12.0 ± 5.99 vs. 9.8 ± 5.33, *p* = 0.001; at re-referral: 11.7 ± 5.77 vs. 9.5 ± 5.81, *p* = 0.010; across both referrals: 12.1 ± 6.44 vs. 9.8 ± 5.74, *p* = 0.036).

### 3.6 The effect of arts on prescription on depression

The same study (22) evaluated the effect of AoP on depression. The results are presented in Table 2 (“Changes in patients’ levels of depression”). Depression symptoms were self-reported using the PHQ-8 before and after the intervention for each referral cycle.

A significant decrease was observed when the assessments were compared between pre- and post-intervention at initial referral (13.4 ± 6.46 vs. 11.5 ± 6.45, *p* < 0.001), at re-referral (13.2 ± 6.23 vs. 10.7 ± 6.21, *p* < 0.001), and between the pre-intervention (initial cycle) and post-intervention (re-referral; 14.0 ± 6.37 vs. 11.28 ± 6.07, *p* < 0.001).

The MCID for PHQ-9 (five points) was not met, and significant differences were observed in the clinical categorisation of the scales (*p* < 0.001; scores 0–4 indicating no significant depressive symptoms, 5–9 indicating mild symptoms, 10–14 indicating moderate symptoms, 15–19 indicating moderately severe symptoms, and 20–24 indicating severe symptoms).

As with anxiety, a decrease in depression symptoms was observed in a multimorbid subgroup, at initial referral (14.3 ± 6.00 vs. 11.7 ± 6.10, *p* < 0.001), at re-referral (13.9 ± 6.12 vs. 10.8 ± 5.57, *p* = 0.002), and when comparing the pre-intervention (initial cycle) and post-intervention (re-referral; 14.5 ± 6.29 vs. 11.2 ± 5.36, *p* = 0.001).

### 3.7 Other relevant effects of arts on prescription

In one study (27), the interruption of the programme was associated with a decrease in wellbeing. The same was observed when participants were re-referred, with increases in anxiety and depression levels and a decrease in wellbeing (22). Sumner et al. (22) observed that the baseline measures of anxiety, depression, and wellbeing were significantly associated with the outcome.

Even though the evaluation focused on wellbeing, Poulos et al. (28) also analysed levels and frequency of creativity, and measures of frailty criteria. No statistically significant differences were observed in the proportion of participants scoring on each of the frailty criteria between pre- and post-intervention. However, a statistically significant increase was observed in both the level of creativity (mean difference = 1.56, 95% CI 1.44–1.98, *p* < 0.001) and the frequency of creativity (mean difference = 1.60, 95% CI 1.06–2.14, *p* < 0.001).

In one study (27), individual mood was self-reported using the SMS, and the participants reported feeling significantly calmer, more relaxed, alert, energetic, content, and well, with mood significantly predicted by time. A larger reduction in tense arousal after art making was associated with increases in global wellbeing over time (*γ* = 0.41, 95% CI 0.07–0.76, *p* = 0.019). Changes in energetic arousal and hedonic tone were not significant predictors.

## 4 Discussion

### 4.1 Summary of main findings

This systematic review synthesised evidence from eight studies on the impact of AoP on wellbeing, depression, and anxiety in adults. Wellbeing improved consistently across all eight studies. Evidence for the reduction of depression and anxiety symptoms derives from a single study (22), in which statistically significant reductions were observed but the minimal clinically important difference was not met for either outcome.

Multimorbid individuals, who have multiple medical conditions and take multiple drugs, have also benefited from AoP programmes (21, 22). Therefore, this intervention could complement existing treatments and provide a more holistic approach to caring for people in these complex situations.

In one study (26), the level of wellbeing after the intervention was relatively low compared with the general population score in Scotland. This could be explained by the fact that the sample had lower levels of wellbeing than the general population, and that the intervention may have been insufficient as a stand-alone treatment for the patients’ condition. The fact that some aspects remained problematic for the participants (26) also highlights the idea that AoP programmes may not be sufficient as a stand-alone intervention.

Participants showed greater engagement when they had more freedom to choose the characteristics of the programme, including art type and location (21). This should be considered when designing and commissioning AoP programmes.

Characteristics such as gender, ethnicity, and baseline scores influenced the outcomes (22, 24). These factors should be considered when developing practical prescribing guidelines. Future studies should investigate additional patient characteristics that may influence outcomes.

### 4.2 Strengths and limitations

No randomised controlled trials (RCTs) were included, and none of the studies had a control group. Therefore, the observed results could be attributable to chance or to other factors rather than the intervention alone.

A further critical limitation is the substantial heterogeneity of both interventions and outcome measures across studies. The AoP programmes included in this review varied considerably in their arts activities (visual arts, music, creative writing, museum-based activities), session frequency, group size, duration (8 to 20 weeks), and facilitator background. This heterogeneity is a recognised challenge in arts and health research: Jensen, Bungay and Holt (37) emphasise that more specific data is necessary to enable the comparison of similar studies. Similarly, the use of three different wellbeing instruments across the included studies (WEMWBS, UCL Museum Wellbeing Measure, MwM-OA), each with distinct conceptual frameworks and psychometric properties, limits the comparability of wellbeing outcomes across studies. The small number of studies analysed, most of which were carried out in the United Kingdom (20–27), could affect the generalisability of the results. Small sample sizes (23, 24, 26) and interventions of short duration are also limitations.

The methodological quality of the included studies was limited by the absence of control groups, limited identification and control of confounding factors, incomplete follow-up, a lack of strategies to address missing data, and a lack of multiple outcome measurements. These limitations should be considered when interpreting the present findings.

### 4.3 Literature gap and further research

Although the results of this systematic review are promising, there is still a significant lack of research in this field. Randomised controlled trials and other rigorous study designs should be developed with the aim of including control groups. This would enable a clearer assessment of causal relationships between the intervention and the outcomes. Additionally, larger and more representative samples should be used to enable the results to be more widely generalised. Future research should also include longer follow-up periods to determine whether the improvements observed at the end of the intervention are sustained over time.

In addition, future studies should consider the identification and control of confounding factors, strategies to minimise loss to follow-up, avoidance of baseline differences between groups, and the inclusion of multiple outcome measurements.

As only one study has investigated the effects of AoP on anxiety and depression, these outcomes should be prioritised in future research.

## 5 Conclusion and future directions

We conclude that AoP programmes are consistently associated with improvements in wellbeing across a range of populations and primary care settings, and that preliminary evidence suggests potential benefits for symptoms of depression and anxiety that warrant investigation in more rigorous study designs. Further studies with more robust designs, larger samples, and longer follow-up periods are needed to better understand the conditions under which AoP is most effective and to inform its implementation in clinical practice and health systems. Referral guidelines should also be developed to help clinicians identify patients who may benefit most from this intervention.

## Data Availability

The original contributions presented in the study are included in the article/Supplementary material, further inquiries can be directed to the corresponding author.
