# Supplementary material for: Arts on prescription for wellbeing in adults: systematic review
Source: Front Public Health. 2026 Jun 1;14:1833798. doi: 10.3389/fpubh.2026.1833798 (PMC13265347; doi:10.3389/fpubh.2026.1833798)
Supplement: Supplementary file 1 [file Data_sheet_1.pdf]

Additional file 1. Search strategies for electronic databases

Pubmed search strategy

#1	Depression [MeSH Terms]
#2	Depressi* [Title/Abstract]
#3	"Depressi* disorder" [Title/Abstract]
#4	"Depressi* illness" [Title/Abstract]
#5	"Depressi* symptoms" [Title/Abstract]
#6	"Depressi* syndrome" [Title/Abstract]
#7	"Emotional depression" [Title/Abstract]
#8	Anxiety [MeSH Terms]
#9	Psychological Distress [MeSH Terms]
#10	Anxiety [Title/Abstract]
#11	"Anxiety disorder" [Title/Abstract]
#12	Psychological Well-Being [MeSH Terms]
#13	Mental Health [MeSH Terms]
#14	Mental Health Recovery [MeSH Terms]
#15	Mental Disorders [MeSH Terms]
#16	Well-being [Title/Abstract]
#17	"Mental health" [Title/Abstract]
#18	"Psychological Wellness" [Title/Abstract]
#19	"Psychological Ill-Being" [Title/Abstract]
#20	"Psychiatric disorders" [Title/Abstract]
#21	Stress [Title/Abstract]

#22	#1 OR #2 OR #3 OR #4 OR #5 OR #6 OR #7 OR #8 OR #9 OR #10 OR #11 OR #12 OR #13 OR #14 OR #15 OR #16 OR #17 OR #18 OR #19 OR #20 OR #21
#23	Art [MeSH Terms]
#24	"Art for health" [Title/Abstract]
#25	"Arts for health" [Title/Abstract]
#26	"Art program*" [Title/Abstract]
#27	"Arts program*" [Title/Abstract]
#28	"Art on prescription" [Title/Abstract]
#29	"Arts on prescription" [Title/Abstract]
#30	"Art referral" [Title/Abstract]
#31	"Arts referral" [Title/Abstract]
#32	"Social prescription" [Title/Abstract]
#33	"Social prescribing" [Title/Abstract]
#34	"Culture prescription" [Title/Abstract]
#35	"Culture referral" [Title/Abstract]
#36	#23 OR #24 OR #25 OR #26 OR #27 OR #28 OR #29 OR #30 OR #31 OR #32 OR #33 OR #34 OR #35
#37	#22 AND #36
#38	"1991"[Date - Publication] : "2024"[Date - Publication]
#39	#37 AND #38

Cochrane Central Register of Controlled Trials (CENTRAL) Search Strategy

#1	MeSH descriptor: [Depression] explode all trees
#2	(Depressi*): ti,ab,kw
#3	("Depressive disorder"): ti,ab,kw
#4	("Depressive illness"): ti,ab,kw
#5	("Depressive symptoms"): ti,ab,kw
#6	("Depressive syndrome"): ti,ab,kw
#7	("Emotional depression"): ti,ab,kw
#8	MeSH descriptor: [Anxiety] explode all trees
#9	MeSH descriptor: [Psychological Distress] explode all trees
#10	(Anxiety): ti,ab,kw
#11	("Anxiety disorder"): ti,ab,kw
#12	MeSH descriptor: [Psychological Well-Being] explode all trees
#13	MeSH descriptor: [Mental Health] explode all trees
#14	MeSH descriptor: [Mental Health Recovery] explode all trees
#15	MeSH descriptor: [Mental Disorders] explode all trees
#16	(Well-being): ti,ab,kw
#17	("Mental health"): ti,ab,kw
#18	("Psychological Wellness"): ti,ab,kw
#19	("Psychological Ill-Being"): ti,ab,kw
#20	("Psychiatric disorders"): ti,ab,kw
#21	(Stress): ti,ab,kw
#22	#1 OR #2 OR #3 OR #4 OR #5 OR #6 OR #7 OR #8 OR #9 OR #10 OR #11 OR #12 OR #13 OR #14 OR #15 OR #16 OR #17 OR #18 OR #19 OR #20 OR #21

#23	MeSH descriptor: [Art] explode all trees
#24	(Art* NEXT for NEXT health): ti,ab,kw
#25	(Art* NEXT program*): ti,ab,kw
#26	(Art* NEXT on NEXT prescription): ti,ab,kw
#27	(Art* NEXT referral): ti,ab,kw
#28	("Social prescription"): ti,ab,kw
#29	("Social prescribing"): ti,ab,kw
#30	("Culture prescription"): ti,ab,kw
#31	("Culture referral"): ti,ab,kw
#32	#23 OR #24 OR #25 OR #26 OR #27 OR #28 OR #29 OR #30 OR #31
#33	#22 AND #32 with Publication Year from 1991 to 2024, in Trials

PsycInfo Search Strategy

S1	MA depression: Search modes - Boolean/Phrase
S2	AB depressi* OR "depressi* disorder" OR "depressi* illness" OR "depressi* syndrome" OR "emotional depressi*" : Search modes - Boolean/Phrase
S3	AB "depressi* symptoms" : Search modes - Boolean/Phrase
S4	MA anxiety: Search modes - Boolean/Phrase
S5	MA psychological distress : Search modes - Boolean/Phrase
S6	AB anxiety OR "anxiety disorder": Search modes - Boolean/Phrase
S7	MA psychological well-being OR mental health : Search modes - Boolean/Phrase
S8	MA mental health recovery : Search modes - Boolean/Phrase
S9	MA mental disorders : Search modes - Boolean/Phrase
S10	AB well-being OR "mental health" OR "psychological wellness" : Search modes - Boolean/Phrase
S11	AB "psychological ill-being" : Search modes - Boolean/Phrase
S12	AB "psychiatric disorders": Search modes - Boolean/Phrase
S13	AB stress : Search modes - Boolean/Phrase
S14	S1 OR S2 OR S3 OR S4 OR S5 OR S6 OR S7 OR S8 OR S9 OR S10 OR S11 OR S12 OR S13 : Search modes - Boolean/Phrase
S15	MA art : Search modes - Boolean/Phrase
S16	AB "art* for health": Search modes - Boolean/Phrase
S17	AB "art* program*" : Search modes - Boolean/Phrase
S18	AB "art* on prescription" OR "art* referral": Search modes - Boolean/Phrase
S19	AB "social prescription" OR "social prescribing" : Search modes - Boolean/Phrase
S20	AB "culture prescription" OR "culture referral": Search modes - Boolean/Phrase

S21	TX "art* on prescription" : Search modes - Boolean/Phrase
S22	S15 OR S16 OR S17 OR S18 OR S19 OR S20 OR S21: Search modes - Boolean/Phrase
S23	S14 AND S22 : Search modes - Boolean/Phrase
S24	PY 1991-: Search modes - Boolean/Phrase
S25	S23 AND S24 : Search modes - Boolean/Phrase
