# Supplementary material for: Arts on prescription for wellbeing in adults: systematic review
Source: Front Public Health. 2026 Jun 1;14:1833798. doi: 10.3389/fpubh.2026.1833798 (PMC13265347; doi:10.3389/fpubh.2026.1833798)
Supplement: Supplementary file 2 [file Data_Sheet_2.pdf]

fronti

Additional file 2. Study Characteristics

Author(s) [Reference]	Referral process	Intervention type	Changes in patients' levels of depression	Changes in patients' levels of anxiety	Changes in patients' levels of well-being	Other outcomes	Other relevant data found
**Crone et al. [20,21]**	Specially designed referral form by their GP or other HP.	The programme was led by an artist in a GP surgery or were based in community facilities. Included activities such as poetry, ceramics, drawing, mosaic and painting. Each activity involved between 3 to 10 patients.	NA	NA	Self-reported Well-being by WEMWBS. A significant increase in WEMWBS scores was observed for participants who attended, those who were engaged and in the multimorbidity subsample, although more modest. Taking all categories of participation into account, a significant increase in WEMWBS scores was also observed.	Participants who were referred to an 8-week programme were more likely to complete it, more engaged, and experienced a greater increase in WEMWBS scores.<br>During the study, the referral process was changed to give patients more freedom to choose some characteristics of the intervention and patients referred after this change showed greater engagement, but there were no statistically significant differences in the rate of change in well-being between patients referred after the change and those referred before the change.	At baseline, those who completed, and those who were classified as engaged reported higher scores on the WEMWBS.<br>There were group differences in occupation, mean number of referral reasons, and the length of the referral course between those who attended and the others.<br>There were also group differences for occupation, and mean number of referral reasons between the "engaged", and "non engaged" groups.
**Thomson et al. [23]**	Referral by a community mental health nurse or day centre.	Combination of outdoor horticultural activities (practical demonstrations and practical activities) and indoor nature-based creative activities (gallery visits, handling of objects, and production of creative responses).<br>Conducted weekly on Tuesdays, the programme was led by a horticultural specialist, an art tutor and a museum volunteer.	NA	NA	Self-reported Psychological well-being by UCL Museum Wellbeing Measure, specifically the positive generic well-being measure.The mean total well-being score had a statistically significant increase. Each of the individual mood items also showed a significant increase, with no significant differences between individual mood items. The greatest improvement was for the word "excited", followed by "inspired".	Themes that emerged from the qualitative phase were building a sense of community, decreasing social isolation and supporting self-esteem.	No one attended all sessions of the programme.

Additional file 2. Study Characteristics

Author(s) [Reference]	Referral process	Intervention type	Changes in patients' levels of depression	Changes in patients' levels of anxiety	Changes in patients' levels of well-being	Other outcomes	Other relevant data found
**Poulos et al. [28]**	Referral by a HP. The participants had to be aged 65 years or older, reside within the catchment area, be able to engage in a small group programme, and had to be either independent or require minimal assistance with self-care.	Courses in the visual arts, photography, dance and movement, drama, singing and music. Each participant could attend a maximum of three consecutive courses (four in exceptional circumstances). The weekly classes, comprised 6 to 8 participants and were led by professional artists with the support of a community care worker or volunteer. Each course concluded with a presentation of work or a performance, and the programme culminated in a professionally curated exhibition.	NA	NA	Self-reported Well-being by WEMWBS. There was a statistically significant increase in WEMWBS scores. 69.5% of the participants showed an increase of three or more points on the WEMWBS. There was no difference in the mean increase in WEMWBS scores according to the number of courses attended.	Measures of frailty criteria were partially self-reported (3 criteria) and programme staff measured the time taken to walk 4 metres at a normal pace and of grip strength in each hand (2 criteria). There were no statistically significant differences between the pre- and post-intervention assessments in the proportion of participants scoring on each of the frailty criteria.<br>Self-reported levels and frequency of creativity by Likert scales. There were statistically significant differences between the pre- and post-intervention assessments for level of creativity and in the frequency of creativity.<br>Open-ended questions were asked about expectations (pre-), how they benefited from a programme, and what they enjoyed the most and the least (post-), using self-report.The courses created a sense of purpose and direction, contributed to a personal growth and achievement, empowered participants and provided a framework for developing meaningful relationships with others.	NA

Additional file 2. Study Characteristics

Author(s) [Reference]	Referral process	Intervention type	Changes in patients' levels of depression	Changes in patients' levels of anxiety	Changes in patients' levels of well-being	Other outcomes	Other relevant data found
**Van de Venter et al. [24]**	Patients with mild to moderate health problems were referred.	The intervention was led by an artist in inner-city GP surgeries or community centers. One group was taken for mothers with babies, while the others were open to all participants. Activities included painting, textiles, music, photography and film.	NA	NA	Self-reported Well-being by WEMWBS. The mean WEMWBS score increased significantly. After a mean of 14 sessions, there was a significant increase in the WEMWBS score.<br>The increase in WEMWBS score was slightly greater for women than for man and for Black and Minority Ethnic participants than for White British participants. Well-being improved more slowly for participants with lower baseline scores.	The model for the association of gender, ethnicity, baseline score and number of sessions attended with outcome WEMWBS scores explained 33% of the variation in outcome WEMWBS scores. After a control for other variables, each additional session attended was associated with a 0.3 increase in WEMWBS, and each point increased in the baseline score was associated with an increase of 0.4 in WEMWBS score.	Only one participant interviewed took antidepressants throughout the intervention period, but stopped taking them a few weeks into her intervention.
**Sumner et al. [22]**	Referral by a HP. Patients could choose from a range of recreative activities, one of which was the programme.	Arts on Prescription intervention programme, which provided up to two referrals for an eight-week arts course. Participants could engage in either visual arts (e.g. painting, ceramics, mosaics, photography) or performing arts (e.g. playwrighting, creative writing, singing) once a week.	Self-reported Depression by PHQ-8. A significant decrease was observed after completion of this AoP programme at initial referral, re-referral and across both cycles. The MCID (minimal clinical important difference) for the PHQ-9 (five points) was not met in a single referral cycle, but significant differences were observed between pre- and post-intervention in clinical categorisation of the scales. The MCID for the PHQ-8 for re-referral or across both cycles was not met, but significant differences in category membership for depression were observed. Analysing the multimorbid subgroup, depression decreased, at initial referral, re-referral and across both cycles.	Self-reported Anxiety by GAD-7. A significant decrease was observed after completion of this AoP programme at initial referral, re-referral and across both cycles.<br>The MCID for the GAD-7 (four points) for initial referral, re-referral and across both cycles was not met, but significant differences were observed between pre- and post-intervention in clinical categorisation of the scales. Analysing the multimorbid subgroup, anxiety decreased at initial referral, re-referral and across both cycles.	Self-reported Well-being by WEMWBS. A significant increase was observed after completion of this AoP programme at initial referral, re-referral and across both cycles.<br>Analysing the multimorbid subgroup, the same was observed.	When participants were re-referred, their pre-intervention levels of each of these variables had almost rebounded to that of pre-initial intervention.	Age, sex, occupation, IMD quintile, number of referral reasons, baseline of well-being, anxiety and depression were considered as potential associations with outcome in the analyses of the data. Only baseline measures of the outcome variable were significantly associated.

Additional file 2. Study Characteristics

Author(s) [Reference]	Referral process	Intervention type	Changes in patients' levels of depression	Changes in patients' levels of anxiety	Changes in patients' levels of well-being	Other outcomes	Other relevant data found
**Thomson et al. [25]**	Health, social care, and third sector organisations referred participants at risk of loneliness and social isolation. Participants had to be able to give informed consent, to travel to the museum, to work in a group, to attend all sessions, take part in interviews, complete questionnaires, not be in work and not regularly attend social or cultural activities. Participants may attend the sessions with a career, friend or family member.	Twelve museum-based interventions comprising a variety of creative and socially interactive sessions including curator talks, behind-the-scenes tours, object handling and discussion, and art activities inspired by the exhibits. Each session lasted approximately two hours and was facilitated by museum staff and volunteers.	NA	NA	Self-reported Psychological well-being by MwM-OA (pre- and post-intervention at start-, mid- and end-programme). Significant participant improvements in all six MwM-OA emotions, pre-post session at start-, mid- and end-programme. 'Absorbed' and 'enlightened', increased disproportionately to the others; 'cheerful' attained the highest pre-post session scores whereas 'active' was consistently lowest.<br>A three-way, 3 × 2 × 6 (factors of programme - start, middle and end; by session- before and after; by emotion), within participants' multivariate analysis of variance (MANOVA) showed a highly significant main effect of programme, session, emotion and a highly significant interaction of session by emotion. A two-way, 3 × 2 (programme by session), within participants' MANOVA showed a highly significant effect of programme and a highly significant effect of session .<br>Comparing the measures of start- and mid-programme, a highly significant difference between pre-session wellbeing scores and a highly significant difference between post-session well-being scores were observed, but no significant differences between mid- and end-programme for pre-session or post-session well-being were showed.<br>All emotions increased highly significantly from pre- to post-session for the start-programme, for the mid-programme and for the end-programme.	NA	NA
**Vogelpoel et al. [26]**	GPs referred older people who experiencing social isolation and associated health problems who had a single or multi-sensory impairment.	Sessions were led by visual and tactile arts facilitators, supported by sense support staff and communicator guides.	NA	NA	Self-reported Well-being by WEMWBS. The overall mean score on the WEMWBS for the group increased by six points (from 41 to 47), indicating a relatively low level of well-being compared to the general population score of 49.9 in Scotland. The number of participants who reporting low well-being decreased from five to three, while the number reporting high well-being increased from one to three. The number of items on the scale with an average score of "3" or more increased from 8 to 12, and positive gains were made in 11 of the 14 areas by the end. The biggest positive change for the group was "feeling more relaxed", but this remained an issue for the participants. Some aspects of well-being decreased very slightly over the programme in the areas of "feeling optimistic", "dealing well with problems" and "feeling good about oneself", but it should be noted that the WEMWBS scores do not consider for the external factors in people's lives.	The qualitative evaluation shows an increased self-confidence, reduced social isolation, establishing new friendships, sense of belonging and group cohesion, mental well-being, art-making, self-value.	WEMWBS scores were collected at the first and last week of the programme for eight of the participants (some were unable to attend the full programme due to illness, appointments or holidays).

Additional file 2. Study Characteristics

**Author(s) [Reference]**	**Referral process**	**Intervention type**	**Changes in patients' levels of depression**	**Changes in patients' levels of anxiety**	**Changes in patients' levels of well-being**	**Other outcomes**	**Other relevant data found**
**Holt NJ [27]**	Participants were usually referred for multiple reasons (mainly for anxiety and depression, social isolation and chronic pain).	Three Arts on Prescription groups were led by skilled arts and health practitioners. Each programme consisted of twelve weekly art workshops, during which participants had access to a range of art techniques and ideas to facilitate their exploration of creativity. They were also provided support to learn and explore new artistic skills.	NA	NA	Self-reported Psychological well-being by WEMWBS (at the start of the programme, before a half-term break, on their return from the break and at the end of the programme). Over the programme, the well-being had a mean increase of 5.01 units (above the minimum of 3 units required for 'meaningful change'). The same was observed for participants at a second programme, with a mean increase of 4.73. The interruption of the programme was associated with a decrease in well-being. Well-being scores changed significantly as a function of time. Some participants did not have the predicted upward slope, but this variation was not statistically significant. Significant changes in well-being were mostly between baseline levels (at the start of the first programme) and subsequent time points (at the end of the first 6 weeks, end of the first programme, end of the second programme). There was also a significant increase in well-being from the beginning to the end of the second programme. None of the decreases in well-being reached statistical significance.	Self-reported Individual mood by SMS (at the beginning and end of each art workshop). Participants reported being significantly calmer and more relaxed, alert and energetic, and content and well. Mood was significantly predicted by time, for all three dimensions: hedonic tone (contentment), tense arousal (relaxation), and energetic arousal (alert). Reporting a larger reduction in tense arousal after art making was associated with increases in global well-being over time. However, changes in energetic arousal and hedonic tone were not significant predictors.	NA

Notes: GAD-7: Generalised Anxiety Disorder Scale. GP: General Practitioner. HP: Health Professional. MwM-OA: Museum Wellbeing Measure for Older Adults. NA: Not applicable. PHQ-8: Patient Health Questionnaire eight-item version. SMS: Short Mood Scale.
